# Reasoning With Conditionals About Everyday and Mathematical Concepts in Primary School

**DOI:** 10.3389/fpsyg.2020.531640

**Published:** 2020-10-29

**Authors:** Anastasia Datsogianni, Beate Sodian, Henry Markovits, Stefan Ufer

**Affiliations:** ^1^Chair of Mathematics Education, Department of Mathematics, LMU Munich, Munich, Germany; ^2^REASON International Doctoral School, LMU Munich, Munich, Germany; ^3^Chair of Developmental Psychology, Department of Psychology, LMU Munich, Munich, Germany; ^4^Department of Psychology, Université du Québec à Montréal (UQAM), Montréal, QC, Canada

**Keywords:** conditional reasoning, primary school ages, domain knowledge, mathematics content, everyday content

## Abstract

A research link between conditional reasoning and mathematics has been reported only for late adolescents and adults, despite claims about the pivotal importance of conditional reasoning, i.e., reasoning with if–then statements, in mathematics. Secondary students’ problems with deductive reasoning in mathematics have been documented for a long time. However, evidence from developmental psychology shows that even elementary students possess some early conditional reasoning skills in familiar contexts. It is still an open question to what extent conditional reasoning with mathematical concepts differs from conditional reasoning in familiar everyday contexts. Based on Mental Model Theory (MMT) of conditional reasoning, we assume that (mathematical) content knowledge will influence the generation of models, when conditionals concern mathematical concepts. In a cross-sectional study, 102 students in Cyprus from grades 2, 4, and 6 solved four conditional reasoning tasks on each type of content (everyday and mathematical). All four logical forms, modus ponens (MP), modus tollens (MT), denial of the antecedent (DA), and affirmation of the consequent (AC), were included in each task. Consistent with previous findings, even second graders were able to make correct inferences on some logical forms. Controlling for Working Memory (WM), there were significant effects of grade and logical form, with stronger growth on MP and AC than on MT and DA. The main effect of context was not significant, but context interacted significantly with logical form and grade level. The pattern of results was not consistent with the predictions of MMT. Based on analyses of students’ chosen responses, we propose an alternative mechanism explaining the specific pattern of results. The study indicates that deductive reasoning skills arise from a combination of knowledge of domain-general principles and domain-specific knowledge. It extends results concerning the gradual development of primary students’ conditional reasoning with everyday concepts to reasoning with mathematical concepts adding to our understanding of the link between mathematics and conditional reasoning in primary school. The results inspire the development of educational interventions, while further implications and limitations of the study are discussed.

## Introduction

The ability to make valid deductions is considered of central importance for scientific reasoning, hypotheses generation, and evaluation (Kuhn et al., 1988), as well as for mathematical thinking (Moshman, 1990; Markovits and Lortie-Forgues, 2011) and learning and success (Nunes et al., 2007; Morsanyi and Szücs, 2014). Crombie (1994) puts forward mathematical deduction as one of six styles of scientific reasoning, which Kind and Osborne (2017) propose as a framework for science education. Indeed, deductive reasoning not only plays an important role for reasoning in mathematics, but it is also a core method to derive scientific hypotheses as conclusions from central assumptions within scientific theories or to draw conclusions from an experiment based on general scientific principles. Kind and Osborne (2017) argue that an exclusive focus of psychological and science education research on a restricted set of scientific reasoning styles, such as experimentation, offers students an “impoverished account of scientific thinking.” In this contribution, we focus on deductive reasoning with mathematical concepts as an important mode of scientific reasoning, which extends and complements research on other scientific styles, such as experimentation.

The term *deductive reasoning* refers to forms of reasoning that ensure that, if the premises are true, the conclusion is necessarily true as well. In particular, in mathematics, deduction is strongly associated with the development of students’ ability to understand formal proofs (Foltz et al., 1995), and it is considered as the central mode of reasoning in mathematical theories. Even basic mathematical concepts are characterized by a strict logical structure, which makes deduction a central mode of reasoning in mathematics at every educational level. One of the key components of deductive reasoning is conditional reasoning, i.e., reasoning with “if–then statements.” Mathematical concepts are characterized by specific properties, which often have the form of conditional statements (e.g., “If the sum of two whole numbers is odd, then their product is even”). Making inferences with such statements requires conditional reasoning skills. Conditional statements are not only a central part of mathematical discourse, but they also occur frequently in everyday language and communication, for example, as with rules such as “If you have a fever, you will have to stay in bed.”

When it comes to conditional reasoning with mathematical concepts, we can assume that knowledge of these concepts develops substantially during primary school. On the contrary, the everyday conditional rules usually applied in research with primary school children (e.g., the fever example above) can be considered to be familiar to them. Of course, detailed knowledge about the causal mechanisms behind these rules reflects substantial knowledge about scientific concepts (e.g., biology in the example above), but the phenomena connected to the rules (having fever, staying in bed, and recovering) are most likely well known to primary school children. For mathematical content (MA), however, we see a parallel development of knowledge about mathematical concepts and connected phenomena, as well as a general improvement in conditional reasoning skills at this age level. It is an open question, however, to what extent conditional reasoning with these contents also requires mathematical knowledge about the concepts involved in the conditional statement. This taps into the question to what extent scientific (and, in particular, conditional) reasoning is guided by general abstract rules that can be applied without much knowledge about the concrete content of the topic at hand and to what extent it relies on explicit knowledge of this content. Conditional reasoning in mathematics has been investigated primarily with adults (Durand-Guerrier, 2003; Stylianides et al., 2004; Inglis and Simpson, 2009; Attridge and Inglis, 2013) and secondary school students, with results suggesting difficulties in reasoning with conditionals in mathematics (e.g., Küchemann and Hoyles, 2002). Despite the potential importance of this form of reasoning, there is very little evidence about elementary students’ abilities to reason with conditionals with MAs. Although previous results have shown that secondary school students have real difficulties with mathematical conditional reasoning (e.g., Küchemann and Hoyles, 2002), psychological studies show that even very young children can reason correctly with conditionals with familiar everyday contents (EDs) (e.g., Markovits and Thompson, 2008). This leads to the question if and how conditional reasoning with mathematical concepts is different from conditional reasoning with EDs.

The present study focuses on primary school students from grades 2, 4, and 6, an age range in which basic mathematical concepts such as arithmetic operations are acquired, which makes it possible to study conditional inferences about these concepts. We will specifically compare elementary students’ conditional reasoning skills about everyday situations and about mathematical concepts. At this age, conditional reasoning skills with everyday contexts have been found in the past (e.g., Markovits and Thompson, 2008; Markovits, 2017), but it is an open question to what extent such skills can be transferred to reasoning about mathematical concepts, which are acquired at this age.

### Conditional Inferences

Conditional reasoning refers to making inferences based on a conditional statement of the form “if *p*, then *q*,” which is called the *major premise* in a conditional reasoning task. In this setting, *p* is called the *antecedent*, and *q* is called the *consequent*. Conditional inferences require a further, *minor premise*. Four different minor premises differentiate four possible logical forms. These four forms can be described systematically by the wording of the minor premise (positive vs. negative) and the type of normative correct conclusion (Table 1). For example, when the major premise is “If the sum of two whole numbers is odd, then their product is even,” a minor premise could be “we have two numbers, which do not have an odd product” (i.e., they have an even product). This premise is negatively worded and allows a definite conclusion: “The sum of the two numbers is not even”). Based on the traditional interpretation of conditionals that *p* is sufficient, but not necessary for *q* (Evans and Over, 2004), and depending on the logical form (minor premise), different conclusions can be drawn. Indeed, one can prove that the sum of two whole numbers being odd is sufficient for their product to be even. However, an odd sum is not necessary to arrive at an even product, as, for example, in 4 × 6 = 24 (even product), but 4 + 6 = 10 (even sum). Definite conclusions are possible for modus ponens (MP; minor premise “*p* is true” or “we have two numbers that have an odd sum” in the example) and modus tollens (MT; minor premise “q is false” or “we have two numbers that do not have an even product”). Acceptance of consequent (AC; minor premise “q is true” or “we have two numbers that have an even product”) does not allow definite conclusions about *p* and *q*. In this case, the conditional does exclude that neither the sum of the two numbers is odd (e.g., 3 and 4), nor it is even (e.g., 2 and 4). Thus, the correct conclusion is indefinite: We cannot say whether the antecedent is true or not. In the same way, denial of antecedent (DA; minor premise “*p* is false” or “we have two numbers that do not have an odd sum”) does not allow a definite conclusion about *q* (or the product of the two numbers).

**TABLE 1:** Logical forms in conditional reasoning for the major premise “if *p*, then *q*.”

Name of form (abbreviation)	Minor premise	Normatively correct conclusion	Minor premise wording	Conclusion type	Minimally required models to make a correct inference
Modus ponens (MP)	“*p* is true”	“so *q* is true”	Positive	Definite	“*p* and *q*”
Modus tollens (MT)	“*q* is false”	“so *p* is false”	Negative	Definite	“*p* and *q*,” “not-*p* and not-*q*”
Affirmation of the consequent (AC)	“*q* is true”	“so *p* or not *p*”	Positive	Indefinite	“*p* and *q*,” “not-*p* and *q*”
Denial of the antecedent (DA)	“*p* is false”	“so *q* or not *q*”	Negative	Indefinite	“not-*p* and not-*q*,” “not-*p* and *q*”

*See Supplementary Tables 1, 2 in the Supplementary Materials (A.1) for explicit examples using conditionals with everyday and MA.*

### MMT of Conditional Inference

One of the most influential theories that have been used to describe conditional reasoning in young children is Mental Model Theory (MMT). This suggests that conclusions are drawn by constructing mental models that encode information about the meaning of the conditional (Johnson-Laird and Byrne, 2002). Such models are generated from a semantic analysis of rules and represent possible states of affairs under these rules. If a given model represents a potential counterexample to a putative conclusion, this conclusion will be denied; otherwise, it will be accepted. Within this perspective, conditional reasoning depends on knowledge about the specific content of a conditional. In other words, individuals use the meaning of premises and their knowledge about the content to think about what is possible given the premises (Nickerson, 2015). Prior studies have shown that MMT accurately describes conditional reasoning processes among elementary students using everyday content (e.g., Markovits, 2000; Markovits and Thompson, 2008). Thus, we will use MMT in the following as the basis for our analyses of conditional reasoning in the primary school age.

According to MMT, humans generate “*p* and *q*” as a standard model for every conditional. To make a valid deduction on MP tasks, an instance of this base model is sufficient: the minor premise *p* is part of the model, and the other part of the model *q* can be used as the correct conclusion. To arrive at a valid conclusion for MT tasks, an additional model “not-*p* and not-*q*” is necessary, because the standard model “*p* and *q*” is not compatible with the minor premise “not-*q*.” To derive that AC does not allow a valid conclusion, minimally models “*p* and *q*” and “not-*p* and *q*” are necessary: Both models are compatible with the minor premise “not-*p*,” but offer different conclusions “*q*” and “not-*q*.” Similarly, the models “not-*p* and not-*q*” and “not-*p* and q” are required for DA inferences. Critical to correctly making indefinite conclusions on the AC and DA forms is the ability to generate models of the form “not-*p* and *q*.” These refer to alternative antecedents, which are counterexamples for the typical errors in AC and DA inferences. Another class of alternative models are instances of “*p* and not-*q*,” which are counterexamples for the correct MP and MT inferences, called disablers (Cummins et al., 1991). Disablers are not compatible with the conditional itself and thus may lead to wrong conclusions. Higher availability of disablers is related to a lower rate of MP and MT acceptances (Cummins, 1995; Janveau-Brennan and Markovits, 1999), whereas higher availability of alternative antecedents is related to higher rates of correct reasoning in AC and DA forms (Markovits and Vachon, 1990; Cummins et al., 1991). Janveau-Brennan and Markovits (1999) found that conditional reasoning in young children (ages 7–12 years) is affected by rates of both alternative and disabler generation in a way that is similar to their effect in adults. In addition, studies indicate that correct DA and AC reasoning correlates negatively with correct MP and MT reasoning (Newstead et al., 2004; Morsanyi et al., 2018). This could reflect a connection between alternative generation (supporting correct DA and AC reasoning) and disabler generation (leading to incorrect MP and MT reasoning).

### Development of Conditional Reasoning

Based on MMT, we can expect the development of conditional reasoning to depend on at least two mechanisms, which will be laid out in the next paragraphs: (1) the acquisition of general schemata to describe conditional statements, which guide adequate interpretation of the different logical forms and provide strategies of model generation independently of the specific content of the conditional, and (2) an increase in knowledge about the conditionals’ contents, which is necessary to build up mental models in general and more specifically to construct disablers and alternatives. While the first mechanism can be assumed to have effects independent of the content of the conditionals, the second mechanism allows for the construction or retrieval of mental models for specific conditionals, which is content dependent.

#### Content-Independent Mechanisms

Within MMT, the general ability to construct interpretations of conditionals that are more complex has been hypothesized to underlie development. Specifically, more complex interpretations require maintaining additional models in memory, which requires increased working memory capacity. According to Barrouillet and Lecas (1999), the development of such general schemata of conditionals starts from the conjunctive-like interpretation (model: “*p* and *q*”), developing to the biconditional interpretation (models: “*p* and *q*”; “not-*p* and not-*q*”) and then to the full conditional interpretation (complete three-model representation: “*p* and *q*”; “not-*p* and not-*q*”; “not-*p* and *q*”). They suggest that the development of conditional reasoning abilities is determined by a developmental increase in working memory capacity. Whereas this model suggests relatively sharp developmental differences, other results show a more gradual change. For example, Janveau-Brennan and Markovits (1999) found a steady age-related development in the ability to make correct AC and DA inferences between grades 1 and 6, as well as a gradual increase in retrieval of disabling conditions leading to less correct MP reasoning. The latter is explained by an erroneous application of disablers to MP inferences. Many studies have found that the AC and DA forms are usually not mastered before the age of 11–12 years, whereas even only about one-third of adults have been found to systematically make these inferences normatively (Gauffroy and Barrouillet, 2009; Ricco, 2010; Moshman, 2011; Markovits, 2014; Christoforides et al., 2016). In addition, many studies (e.g., Janveau-Brennan and Markovits, 1999) have found that specific content strongly affects conditional reasoning. Summarizing, the current evidence indicates that, possibly connected with working memory capacity, children acquire schemata of conditional reasoning, which allow correct MP reasoning first, then MT, and later AC and DA reasoning. However, as we shall see, there are clear indications that, because of the necessity to retrieve or generate alternatives and disablers, these reasoning skills are subject to important content effects.

Regarding the development of conditional reasoning with positively versus negatively worded minor premises (i.e., MP and AC vs. MT and DA), the literature provides less information. The negations involved in negatively worded minor premises have been hypothesized to pose specific difficulties (Schroyens et al., 2001). MMTs usually assume that mental models only represent *possibilities* that can occur given the premises—not what is impossible given the premises (principle of truth, Johnson-Laird, 2001), which could lead to problems if a negation leads to an unspecified situation (e.g., while “not wet” has a similar meaning as “dry,” “blue” has no specific opposite standing for “not blue”). The latter case would require an abstract understanding of negations and thus can be seen as a content-independent part of conditional reasoning skills. However, the account for the effects of positive versus negative wording in the literature is not as explicit as for definite versus indefinite forms.

#### Content-Specific Mechanisms and Knowledge About Conditionals’ Contents

Apart from general individual development, the specific content of the conditional has been found to influence the ability to make conditional inferences. Previous studies (Markovits, 2000; Markovits and Thompson, 2008) have shown that even 6- or 7-year-old children can reason logically with the AC and DA inferences, when the content refers to simple categorical premises (e.g., “If an animal is a cat, then it has legs”). In particular, in the study by Markovits and Thompson (2008), 6-, 7-, and 9-year-old students were observed to make valid conclusions about MP and AC inferences in a familiar categorical context (e.g., “If something is a car, then it has a motor. Now, suppose that you see a car” under either categorical instructions (“Is it certain that it has a motor?”) or probabilistic instructions (“How sure is it that it has a motor?”). Chao and Cheng (2000) also found evidence of the domain specificity of conditional reasoning in preschool children. In detail, the latter study examined preschoolers’ conditional reasoning skills within permission and arbitrary concepts showing that pragmatic (permission) conditional rules (e.g., “If it is windy, then he must not have shorts on”) seem to emerge earlier than formal (arbitrary) conditional rules (e.g., “If it is windy, then there must not be an orange in the box”) as MP and MT inferences were approached reliably by the students only in the permission context. These kinds of effects have led to the conclusion that conditional reasoning might be domain-specific (e.g., Cummins, 1996b; Chao and Cheng, 2000), especially in early ages. In fact, based on a series of studies by Markovits and Lortie-Forgues (2011) and Markovits (2014), which examined the context effects as well as the alternative generation effects on conditional reasoning skills among individuals from age 7 to age 19 years, a clear developmental pattern was proposed. This pattern (Markovits, 2014) suggests that 7- and 8-year-old students possess conditional reasoning skills with categorical premises, 10- to 12-year-old children can make logical conditional inferences with familiar causal premises; 14–16 year olds can do so with causal and counterfactual premises, whereas adults older than 20 years also perform well with abstract conditionals.

That conditional reasoning performance depends on the content of the conditionals is in line with core assumptions of MMT. According to this, mental models need to be retrieved or constructed based on knowledge about the situation contained in the conditional statement. As previously mentioned, studies on the effects of content on conditional reasoning have concentrated on broad categories, which have been shown to affect in particular retrieval of alternative antecedents (e.g., Markovits and Lortie-Forgues, 2011). However, the effects of more specific forms of content variation, such as that involved in reasoning with mathematical concepts, have not often been studied.

### Conditional Reasoning About Mathematical Concepts

One possible type of content, for which knowledge is acquired during primary school age, comprises mathematical concepts. Consider, for example, the conditional “If a house has three floors with four windows each, then it has 12 windows.” Representing this situation *per se* does not require substantial mathematical knowledge beyond representing cardinal numbers, which is usually acquired by early primary school age (e.g., Litkowski et al., 2020). For example, representing a model of the type “*p* and *q*” would consist of an instance of a house that has three floors with four windows each. Calculating that there are 12 windows overall is a basic procedure, which can be solve by primary school students with a variety of basic strategies (e.g., addition 4 + 4 + 4, or fact retrieval of 3 × 4 = 12). However, generating an explicit alternative would involve imagining a different configuration of floors and windows per floor, which does consist of 12 windows (e.g., two floors with six windows each). This involves solving a reverse task, specifically to find a different configuration (e.g., two floors with six windows each) that also leads to 12 windows overall. Finding pairs of factors that have a given product requires substantial mathematical knowledge about multiplication, which has been found to develop slowly even until the end of primary school (e.g., Robinson et al., 2018).

Only sparse evidence about conditional reasoning with mathematical concepts is available for primary school. The mathematics education literature has focused mostly on older learners and has shown, for example, that dealing with mathematics and participating in mathematics instruction can lead to improved conditional reasoning skills (Handley et al., 2004; Inglis and Simpson, 2008, 2009; Attridge and Inglis, 2013; Morsanyi et al., 2017; Morsanyi et al., 2018). For secondary school students, research has focused mainly on conceptual issues such as the differentiation between a statement and its converse and less on drawing conditional inferences (Küchemann and Hoyles, 2002).

Given that both general schemata for conditional reasoning as well as mathematical knowledge develop during primary school age, it is of substantial interest to understand how these two developments interact. It can be assumed that knowledge about the content of familiar conditionals is widely available at this age, whereas knowledge about mathematical concepts varies substantially (Robinson et al., 2018). This would suggest that reasoning with mathematical concepts would be more difficult than reasoning with everyday statements. This would be consistent with prior results showing early reasoning skills with everyday content (e.g., Markovits and Thompson, 2008) and reports on secondary school students’ problems dealing with mathematical conditionals (Küchemann and Hoyles, 2002). Different mechanisms could be hypothesized to explain such differences. First, the mathematical content of the conditional might affect decoding and representation of the conditional, which would be reflected in a relatively coherent performance difference between everyday reasoning and reasoning with mathematical content over all logical forms. This effect could also be moderated by the development of mathematical knowledge during primary school age and thus be larger for younger students. Beyond the initial problem representation, the retrieval or construction of alternative mental models is a second point in the MMT account of conditional reasoning that is particularly dependent on content-related knowledge. MMT would predict stronger content-related differences here for the two indefinite forms AC and DA, because more models are required to make valid inferences on these forms than for the definite forms MP and MT. Indeed, prior research has found an influence of alternative generation skills on AC reasoning rather than on MP reasoning (Janveau-Brennan and Markovits, 1999; Markovits and Quinn, 2002). Finally, because this effect depends on available knowledge about the conditional’s content, it should be more pronounced for students in lower grades, leading to an interaction of content, logical form, and grade level. Given the lack of available evidence on elementary school students’ conditional reasoning skills with mathematical concepts, more substantial hypotheses are hard to derive. However, the role of alternative generation found in prior studies with elementary school students (e.g., Markovits, 2017), together with progress in mathematical knowledge during elementary school age (Robinson et al., 2018), speaks to expecting the interaction of content, logical form, and grade level.

### The Current Study

In this study, we contrast conditional reasoning about premises involving mathematical concepts with reasoning about familiar causal premises. To this end, we study conditionals about easily accessible situations that contain mathematical structures (e.g., numbers of windows in configurations for floors and windows per floor as in the example above). We chose structures related to multiplication and addition, concepts that are introduced in the first years of primary school. We assume that increasing knowledge about these concepts will affect conditional reasoning performance on top of the well-described development of conditional reasoning skills with familiar premises.

The main goal of this study is to investigate to what extent reasoning about mathematical concepts specifically affects primary school students’ conditional reasoning in the four different logical forms and its development. Beyond replicating findings on the development of logical reasoning with familiar everyday statements, the following questions are addressed:

(1a) Is there a general disadvantage of reasoning with mathematical content (MA), compared to everyday content (ED), for primary age students?

Understanding the situations, in which we embedded the mathematical content for the conditionals, did not require substantial mathematical knowledge. Thus, we did not put forward a specific hypothesis about whether this factor would show a main effect.

(1b) Is such a general disadvantage larger for children in lower grades, as compared to upper primary school grades?

Again, because we embedded the mathematical concepts and structures in easily accessible situations, we also did not put forward a specific hypothesis about this specific interaction.

(2a) Is there a specific disadvantage of reasoning with mathematical concepts, as compared to everyday conditionals, for the indefinite logical forms AC and DA?

The role of alternative generation for reasoning with indefinite forms has been shown for everyday reasoning. Moreover, generating alternatives for the mathematical structures reflected in our conditionals requires well-connected mathematical knowledge. Thus, we expected a corresponding interaction of content and the type of inference (definite for MP and MT vs. indefinite for AC and DA).

(2b) Is there a disadvantage of a negative (vs. positive) wording on conditional reasoning on both contexts, for definite and indefinite conditionals?

Given the sparse evidence on the effects of positive versus negative wording, we approached this question in an exploratory manner, expecting lower performance for negative wordings because of the specific difficulties involved in dealing with negations.

(2c) Are the effects under (2a) and (2b) dependent on students’ grade level?

Because knowledge about additive and multiplicative structures develops over primary school age, we expected that the effect discussed under (2a) would be more pronounced for students in lower grades, as compared upper primary school grades.

## Materials and Methods

### Participants

Around 300 students and their parents were approached for participation in this cross-sectional study. A total of 102 elementary students (average age, 10 years 1 month) from grades 2, 4, and 6 living in Cyprus participated. Regarding the participants’ socioeconomic background, the median category on the “books at home” question (Paulus, 2009) was “one complete bookcase (26–100 books)” in all grades, and distribution over the five answer alternatives (from “no or very few books” to “over 200 books”) did not differ significantly over grades [χ^2^(8) = 13.4, *p* = 0.10]. Parents’ written consent and children’s oral assent were obtained for all participants. Further information about the sample is displayed in Table 2.

**TABLE 2:** Sample size, mean, and standard deviation (SD) of age in years and working memory scores by grade level.

Grade	*n*	Age in years, mean (*SD*)	Working memory score, mean (*SD*)
2	32	8.22 (0.72)	2.16 (0.99)
4	33	9.94 (0.53)	3.18 (1.04)
6	37	11.82 (0.53)	4.19 (1.66)

### Design

Each participant took part in one individual 45- to 60-min face-to-face interview during regular school hours with the first author in a separate room of the school. The factors relating to *content* (everyday vs. mathematical) and logical form (*positive vs. negative* wording of minor premise, *definite* vs. *indefinite* conclusion, and the interaction of the two) were varied within subject, with randomized sequence of two content blocks, randomized sequence of conditionals within each content block, and randomized sequence of four minor premises (each relating to a different logical form) within each conditional.

#### Procedure

Initially, students were asked for their age, native language, and approximate number of books at their house (“books at home” question; Paulus, 2009). Participants were clearly informed about the anonymity and confidentiality of their replies, as well as their voluntary participation, clarifying that they were free to withdraw from the interview process anytime without any negative consequences. Then, participants were familiarized with the three answer alternatives (“yes, this is certainly so,” “no, this is certainly not so,” “you cannot say for sure, whether it is so, or not”) and shown how to select their answers on the tablet computer screen. The answer options were represented by symbols on the tablet screen: A green check mark for “yes, this is certainly so,” a red “x” for “no, this is certainly not so,” and a question mark for “you cannot say for sure, whether it is so, or not”. A short game with three questions about hidden marbles was used to check for comprehension of these answer alternatives (see Supplementary Materials A.3). An explanation was given in case of wrong answers. Afterward, two blocks (everyday vs. mathematical) of four conditionals each were presented (10–15 min per block). Each conditional was presented separately, and students were asked to make four conclusions based on four different minor premises, corresponding to the four logical forms. Then, a block of alternative generation tasks followed, which is not examined in this article. In the end of the interview, students’ responses to a short working memory test and an arithmetic calculation test (not examined in this paper) were gathered.

All tasks were displayed using a tablet-based interview system, and children were expected to select their preferred answer by touching the screen on the respective part of the visual representation of answer alternatives. The interview system also randomized the sequence of blocks, conditionals, and minor premises. By ensuring this full randomization of the questions’ order, we systematically controlled for possible order effects. No justifications for the answers were requested as we were interested in students’ intuitive responses.

##### Conditional reasoning tasks

Eight conditionals were used to measure conditional reasoning skills (four conditionals per content condition). The verbal structure of the tasks was parallel in both content blocks. All conditionals were presented verbally and in a written form on a tablet computer. Participants were told for each conditional that they should assume that it was really true. For each of the four conclusions to be made on each conditional, students were presented with the major and the minor premise verbally and on the screen. They were asked if they could conclude that a given conclusion was *true for sure*, if it was *not true for sure*, or if *no definite conclusion* was possible. For example, for the conditional “If someone’s finger is cut deeply while cooking, then it bleeds,” the minor premise “George’s finger is not bleeding” would have been presented to test the logical form MT. The students would have been asked, “Based on what he knows, what can Peter [the central character in our cover story] say for sure?” and the answer options were “George’s finger has just been cut deeply while cooking” (yes), “George’s finger has not just been cut deeply while cooking” (no), and “He cannot be sure whether George’s finger has just been cut deeply while cooking or not” (uncertain).

The everyday conditional reasoning tasks contained familiar causal conditionals (with the antecedent and the consequent being the cause and effect, respectively: If a glass is dropped in the kitchen, then there is a sound; if someone’s finger is cut deeply while cooking, then it will bleed; if someone jumps into a pool, then they will get wet; if someone breaks their arm, then they will hurt). The verbal structure of the introduction, conditionals, and answer alternatives were based on previous studies on conditional reasoning (e.g., Markovits and Lortie-Forgues, 2011).

The conditionals with mathematical content dealt with situations that contained mathematical structures. The specific structures and related concepts were multiplication and addition, because these concepts are included in the national curriculum up to grade 2. Comprehension questions were included for the conditionals, to control if students understood the situation in which the major rule was embedded.

For example, one of these situations was introduced in the following way:

“Peter is walking with the little explorers, and they just found some treasure boxes. We know that the boxes contain some blue and red diamonds. Each blue diamond is worth three gold coins. Each red diamond is worth two gold coins.”

The corresponding comprehension question was: “In a treasure box, there is one blue diamond and two red diamonds. How many gold coins is this worth?” In case the child provided the correct answer (7), the reasoning tasks followed. After a wrong answer, the researcher repeated the explanations and posed the comprehension question for a second and last time. The answer was recorded, and the researcher continued without providing any feedback or hints to students. For mathematical conditionals, only answers on reasoning tasks for which the corresponding comprehension question was answered correctly were included in the analyses. In grade 2, answers to 62 of 128 presented conditionals (48.4%) were excluded, with eight students being excluded on all conditionals. In grade 4, 26 of 132 conditionals (19.7%) were excluded, and in grade 6, 15 of 148 conditionals (10.1%). In grades 4 and 6, at least two conditionals were included for each student.

The conditional (major rule) in the example before was “If the box contains exactly two blue diamonds and three red diamonds, then the diamonds in the box are worth 12 gold coins. It is certain that this is really true.” For example, the logical form MT was presented in the following way: “This is Stelios. The diamonds in his box are not worth 12 gold coins. Based on what he knows, what can Peter say for sure?” The alternative answers were parallel to the everyday conditionals: “The box contains exactly two blue diamonds and three red diamonds” (yes), “The box does not contain exactly two blue diamonds and three red diamonds” (no), and “He cannot be sure whether the box contains exactly two blue diamonds and three red diamonds or not” (uncertain).

Overall, the students worked on 32 conditional reasoning tasks (2 content types × 4 conditionals each × 4 logical forms each). All tasks were tested through a pilot study ensuring their appropriateness for this age range (Datsogianni et al., 2018).

##### Working memory test

Working memory capacity was chosen as a control variable, because it has been found to predict mathematics skills (Holmes and Adams, 2006), as well as logical reasoning skills (Nakamichi, 2007, 2011). To measure working memory capacity, a backward digit span test (Wechsler Intelligence Scale for Children IV Digit Span Subtest) was used, as in previous studies on young children’s conditional reasoning skills (e.g., Nakamichi, 2007, 2011). Specifically, sequences of digits were read out loud, and students were asked to repeat them in the reversed order. In the beginning they were provided with an example of a sequence of three digits (e.g., 9–2–7) and were asked to reproduce it backward; in case of a correct reply, the test continued. In case of a wrong reply, the correct response was given (7–2–9), and then a second example was presented. Regardless of the reply to the second example, no feedback on this was given. The test consisted of 14 trials. The first two trials contained two digits, each (2–5, and 6–3). The number of digits increased by one after every second trial. The test was discontinued after failure on two trials with the same number of digits. No hints were given on any of the trials. Each trial was scored 2 (if the child passed both trials), 1 (if the child passed only one item), or 0 (if the child failed both trials).

##### Analyses

The answers to the conditional reasoning tasks were analyzed using generalized linear mixed models, a generalization of logistic regression. It allows analyzing the data on item level, but still takes into account dependencies between answers provided by each student and on each task. The package lme4 in R was used (Bates et al., 2015). Grade level (grades 2, 4, and 6) was included as a between-subject factor, and content (everyday vs. mathematical), wording of the minor premise (positive vs. negative), and type of conclusion (definite vs. indefinite) as within-subject factors. Wald χ^2^ tests were used to compare models during model selection and to analyze omnibus effects. Planned contrasts of estimated marginal means were used to compare performance in different cells of the experimental design. These contrasts are expressed in logarithmic odds ratios. For example, a main effect contrast of *b* = 0.50 can correspond to differences in solution rates of up to

$$\frac{e^{0.77+\frac{0.50}{2}}}{1+e^{0.77+\frac{0.50}{2}}}-\frac{e^{0.77-\frac{0.50}{2}}}{1+e^{0.77-\frac{0.50}{2}}}=10.8\%$$

around the mean solution rate of the items in our study (a logit of 0.77 corresponds to the mean solution rate of 59.5% in our study). However, corresponding differences in solution rates can be substantially smaller, when very easy or very difficult task variants are compared. Bonferroni correction was applied when analyzing multiple contrasts along the same factors.

##### Ethics statement

The ethics approval was obtained from the Centre of Educational Research and Evaluation of Cyprus Pedagogical Institute, as well as the Cyprus Ministry of Education and Culture. Back-and-forth professional translation, from the original English language of the interview protocol into the Greek language and back, was conducted. Parents and students were informed that the participation in the study was completely voluntary, that answers would be handled confidentially, and that they could stop their participation at any time without any further consequences.

## Results

### Descriptive Results

**FIGURE 1:**
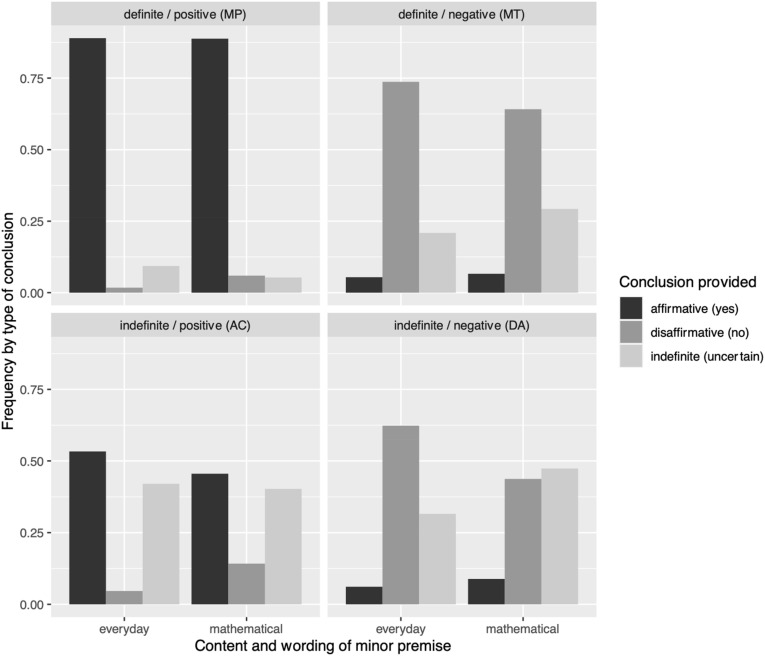
Frequency of answers given by conditional content and logical form. ED, everyday content; MA, mathematical content.

Overall, 59.5% of students’ answers in the conditional reasoning tasks were correct. Figure 1 shows the frequency of each answer option by conditionals’ content and logical form. MP tasks were mostly answered correctly with affirmative conclusions (“*q* is true”), and correct disaffirmative conclusions (“*p* is false”) were observed in more than 50% of the answers to MT tasks. Wrong indefinite conclusions (“uncertain”) occurred rarely for MP tasks, but more often for MT tasks. For AC tasks, the correct indefinite conclusions (“one cannot say whether *p* is true or not”) were about as frequent as the wrong affirmative conclusions (“*p* is true”). For DA tasks, correct indefinite conclusions (“one cannot say whether *q* is true or not”) occurred less often than wrong disaffirmative conclusions in the everyday context, while the frequencies were comparable for the mathematical context. Summarizing, performance was highest in MP tasks (correct answer affirmative), followed by MT tasks (correct answer disaffirmative), whereas AC and DA tasks showed descriptively similar frequencies of correct indefinite conclusions (“uncertain”), with a slightly lower performance on everyday DA tasks.

For all combinations of grade levels, logical forms, and contents, the distribution of responses over the three possible options (affirmative, disaffirmative, and indefinite) differed significantly from a uniform distribution in all conditions, except for grade 2 on items with MA and the two negative forms (Bonferroni correction for 24 tests, *p* = 0.02 for MT and *p* = 0.10 for DA). Thus, that students might have applied a systematic guessing strategy must be taken into account for these two conditions.

### Model Selection

In an initial step of model selection, we decided on the random intercepts and slopes to be included. Given the low number of conditionals per person, we analyzed only the random slopes for main effects of grade level and logical form over the different conditionals. Initial analyses indicated that including random slopes for interaction effects over individuals lead to singular model fit. Because singular models are prone to misinterpretation (Bates et al., 2018), we decided to analyze only random slopes for main effects of content and logical form over individuals. χ^2^ difference tests indicated that leaving out the random slope for grade level over conditionals from a model containing random intercepts and random slopes for all main effects over conditionals and persons did not affect model fit significantly. However, removing the random slopes for the remaining main effects over persons and removing the random slopes for the remaining main effects over conditionals each affected model fit significantly. Thus, we decided to select the model with random intercepts and random slopes for *wording of the minor premise* (positive vs. negative) and *type of conclusion* (definite vs. indefinite) over conditionals, as well as random intercepts and random slopes for *wording of minor premise*, *type of conclusion*, and *content* over individuals. In a last step, we removed all non-significant interactions of fixed effects, which were not part of other significant interactions. This did not reduce model fit significantly, as well. Results of the χ^2^ difference tests and the lme4 formulas for the models considered during model selection are given in the Supplementary Materials A.2.

**FIGURE 2:**
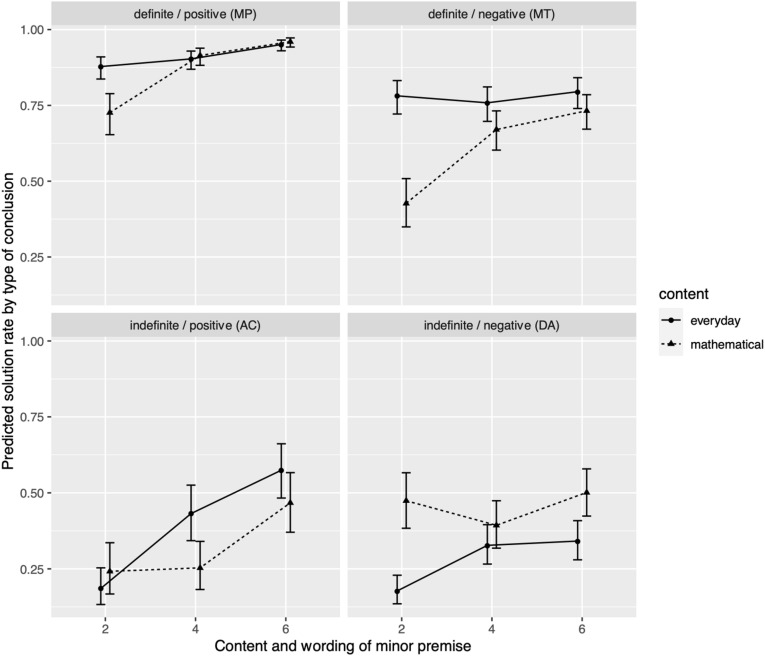
Predicted solution rates and standard error of estimated marginal means (prediction and prediction ± 1 standard error, transformed to the 0-to-1 scale for solution rates) of students’ conditional reasoning scores by grade level, logical form, and conditionals’ content.

χ^2^ statistics for the fixed main and interaction effects in the final model are given in Table 3. Working memory as a control variable did not predict conditional reasoning scores significantly (Table 3). Estimated marginal means and confidence intervals are available in Figure 2.

**TABLE 3:** χ^2^ statistics for the fixed main and interaction effects in the final model, in the order of occurrence in the analysis section.

Relates to question	Fixed effect	df	χ^2^(df)	*P*	
	Working memory	1	2.24	0.13	
1a	C: content (everyday vs. mathematical)	1	0.92	0.34	
1b	G: grade level	2	24.11	<0.001	***
1b	G × C	2	0.69	0.71	
2a	W: wording of minor premise (positive vs. negative)	1	5.83	0.02	*
2a	T: type of correct conclusion (definite vs. indefinite)	1	94.65	<0.001	***
2a	W × T	1	39.34	<0.001	***
2a	C × W	1	1.12	0.29	
2a	C × T	1	9.92	<0.01	**
2a	C × W × T	1	15.57	<0.001	***
2b	G × W	2	10.64	<0.01	**
2b	G × T	2	0.01	0.99	
2b	G × C × T	2	22.01	<0.001	***

*p < 0.05, **p < 0.01, ***p < 0.001.

### General Content Effect (Question 1a)

Students answered 59.1% of the questions with ED and 60.2% of the questions with MA correctly. The main effect of *content* was not significant (Table 3).

### Grade-Dependent Content Effect (Everyday vs. Mathematical, Question 1b)

The main effect of grade level was significant (cf. Table 3). Over both content conditions, students from grade 6 made significantly more correct inferences than students from grade 4 (67.0% vs. 59.3%, planned contrast *b* = 0.51, *p* < 0.01) and students from grade 4 made more correct inferences than students from grade 2 (59.3% vs. 49.2%, planned contrast *b* = 0.54, *p* < 0.01). The interaction between *content* and *grade level* was not significant (Table 3), providing no evidence of a grade-dependent effect of content on conditional reasoning in general (i.e., over all logical forms).

### Grade-Independent, Form-Specific Content Effects (Question 2a)

The main and interaction effects referring to logical form (*wording* of minor premise, *type* of conclusion, and their interaction) were significant (Table 3). In line with prior results from the everyday contents, a planned contrast analysis showed significantly more correct MP inferences (88.9% of all MP inferences) than MT inferences (69.6%, *b* = 1.41, *p* < 0.001) and significantly more correct MT than AC (41.3%, *b* = 1.50, *p* < 0.001) and DA (48.4%, *b* = 1.44, *p* < 0.001) inferences. Performance of DA and AC inferences did not differ significantly (*b* = 0.06, *p* = 1.00). This indicates that the main effect of *conclusion type* (more correct definite than indefinite responses) was modulated by *wording* of the minor premise only for the definite forms, indicated by lower MT (negative wording) compared to MP (positive wording) performance.

Among the interaction effects with the *content* factor, the interactions with *type* of conclusion (definite vs. indefinite), as well as the three-way interaction of *content*, *type* of conclusion, and *wording* of the minor premise (positive vs. negative) were significant (Table 3). For both contents, fewer indefinite correct conclusions were drawn than definite correct conclusions (ED: 36.8% vs. 81.3%, *b* = 5.10, *p* < 0.001; MA: 43.8% vs. 76.4%, *b* = 3.61, *p* < 0.001). Contrary to our expectations, this effect was significantly more pronounced with everyday than with mathematical contents (*b* = 1.49, *p* < 0.01).

A closer analysis indicated that (averaged over all grade levels) there were no significant content-related differences for the two forms with positive wording of the minor premise (MP, positive/definite: 89.0% in ED vs. 88.8% in MA, *b* = 0.21, *p* = 1.00; AC, positive/indefinite: 42.0% in ED vs. 40.3% in MA, *b* = 0.72, *p* = 1.00). However, there were marginally more correct MT (negative/definite) inferences with everyday than with mathematical content (73.7% in ED vs. 64.1% in MA, *b* = 0.79, *p* = 0.06) and significantly fewer correct DA (negative/indefinite) inferences with everyday than with mathematical content (31.5% in ED vs. 47.4% in MA, *b* = −0.79, *p* < 0.05). This indicates that the weaker difference between definite and indefinite forms for mathematical, compared to everyday content, was mostly due to the two forms with negative wording. It seems to be mainly caused by better DA (indefinite) and (marginally) lower MT (definite) reasoning with mathematical, compared to everyday content.

### Grade- and Form-Dependent Content Effects (Question 2b)

Two significant interactions were observed with a connection to this question. First, as a preliminary result, a significant interaction between *grade level* and *wording* (positive vs. negative) of the minor premise occurred (Table 3). Such an effect had not been anticipated because of scarce evidence on the effects of positive versus negative wording of minor premises. A significant performance difference in reasoning with positively worded logical forms over the grade levels could be observed (grade 2: 52.7%, grade 4: 64.6%, grade 6: 74.2%; grade 2 vs. grade 4: *b* = 2.92, *p* = 0.02, grade 4 vs. grade 6: *b* = 3.05, *p* < 0.01). For negatively worded logical forms, only a significant difference between grades 2 and 6 could be found (grade 2: 45.8%, grade 4: 54.0%, grade 6: 59.7%, grade 2 vs. grade 4: *b* = 1.36, *p* = 0.44, grade 4 vs. grade 6: *b* = 1.01, *p* = 0.83, grade 2 vs. grade 6: *b* = 2.37, *p* = 0.03). Even in grade 6, performance on negatively worded logical forms was significantly lower than on positively worded forms (*b* = 4.59, *p* < 0.001), and indeed, differences between grades 2 and 6 were significantly stronger for positively than for negatively worded forms (*b* = 3.61, *p* < 0.01). This speaks for a slower development of reasoning with negatively worded, as compared to positively worded, minor premises.

Moreover, the three-way interaction of *grade level*, *content*, and *conclusion type* (definite vs. indefinite) was significant (Table 3). Averaging over all grades, having to draw an indefinite conclusion had already turned out to have a smaller negative impact on reasoning performance with mathematical than with everyday content (see question 1b). Contrast analyses revealed that, for all grades and both content types, except for mathematical content in grade 2, questions with indefinite correct conclusions lead to lower performance than questions with definite correct conclusions (Table 4). Moreover, these contrasts differed significantly between the two contents only in grade 2 (Table 4). This indicates that the content-dependent effect of definite versus indefinite conclusions was caused mostly by a stronger difference for everyday, compared to mathematical content, in grade 2.

**TABLE 4:** Solution rates and contrasts between definite and indefinite forms by grade level.

Grade	Content	Solution rates				Definite/indefinite contrast		Content contrast	
		MP	MT	AC	DA	*b*	*p*	*b*	*p*
2	ED	85.9%	70.9%	21.1%	21.9%	6.26	<0.001	4.33	<0.001
2	MA	71.6%	44.8%	30.3%	44.8%	1.93	0.15		
4	ED	86.4%	73.5%	46.2%	34.1%	4.37	<0.001	−0.22	1.00
4	MA	88.6%	67.9%	36.8%	40.6%	4.59	<0.001		
6	ED	93.9%	76.4%	56.5%	37.7%	4.67	<0.001	0.36	1.00
6	MA	97.8%	71.0%	48.1%	54.2%	4.31	<0.001		

*The content contrast is the difference of the two definite/indefinite contrasts for the respective grade level.*

In particular, this pattern is reflected in better DA reasoning with mathematical than with everyday content in grade 2 (*b* = −1.43, *p* < 0.01). However, this was combined with significantly lower MT reasoning performance with mathematical as compared to everyday content in grade 2 (*b* = 1.57, *p* < 0.001; for MP: *b* = 1.00, *p* = 0.11; for AC: *b* = −0.34, *p* = 1.00).

### Exploratory Analyses of Provided Answers

To explain the observed pattern of effects, in particular the better reasoning performance with mathematical compared to the everyday content for indefinite conclusions in grade 2, we analyzed how often students chose the indefinite “uncertain” answer (Table 5). We hypothesized that the specific pattern of higher DA and lower MT reasoning could be due to a stronger tendency to give an uncertain response when reasoning about mathematical content, possibly due to difficulties in retrieving or constructing a representation of the problem situation when dealing with the negative forms MT and DA. The amount of indefinite answers increased descriptively from grade 2 to grades 4 and 6 for EDs (Table 5). For mathematical content, it was already rather high in grade 2 and remained on this level in grade 4 and grade 6. In grade 2, significantly more indefinite responses were provided with mathematical than with everyday content. In a similar vein, the stronger differences between the two conclusion types by wording of the minor premise (see analyses for question 2a) have to be seen in the context of significantly more indefinite responses on negatively worded questions with mathematical (38.3%) than with everyday content [26.2%, χ^2^(1) = 23.18, *p* < 0.001], whereas there was no significant difference on positively worded questions [MA: 22.7% vs. ED: 25.6%, χ^2^(1) = 1.44, *p* = 0.23].

**TABLE 5:** Frequencies of indefinite (“uncertain”) responses by grade level and content, and χ^2^ tests for content differences.

Grade	ED	MA	χ^2^(1)	*p*
2	18.6%	31.5%	15.7	<0.001
4	28.0%	27.9%	0.0	1.00
6	30.4%	32.2%	0.34	0.56

## Discussion

The main goal of this study was to investigate students’ reasoning with conditionals about mathematical concepts, which are still emerging during primary school. To this end, reasoning with MAs was contrasted against reasoning with familiar causal premises with EDs. Based on MMT, we started from the assumption that content differences could arise due to general difficulties representing the situations in which the conditionals with MA were embedded, or from specific problems generating alternative models based on these contents. We primarily assumed the latter, because representing the embedding situations for our conditionals did not require specific mathematical knowledge (and we controlled for comprehension of the situations), whereas constructing or retrieving alternatives was strongly contingent on such prior knowledge. Finally, we assumed that both effects could be modulated by the increase in mathematical knowledge during primary school age, leading to more pronounced content effects in earlier compared to later primary school grades. Given the sparse evidence, we did not put forward hypotheses regarding positive or negative wording of the minor premise.

### Overall Performance

Considering average performance over all logical forms and grades, more than 50% of students’ replies were correct, which is substantially above a guessing probability of 33.3%. This could be taken as evidence in favor of the claim that elementary students do possess early conditional reasoning skills to some extent (e.g., Markovits and Thompson, 2008). In line with previous studies (e.g., Klaczynski and Narasimham, 1998; Markovits and Barrouillet, 2002; Klaczynski et al., 2004; Gauffroy and Barrouillet, 2009), the results indicate that students’ performance increases with grade level. Moreover, in line with prior research (e.g., Barrouillet and Lecas, 1999), we found that MP reasoning was easier for elementary school students than MT reasoning, which was in turn easier than AC and DA reasoning, averaging over both contexts and logical forms (Markovits et al., 1996; Markovits, 2000). However, our results did not completely match our predictions made on the basis of MMT.

### General Content Effects

We did not find a general effect of content, averaging over all logical forms and grade levels. This indicates that conditionals with mathematical content did not pose general difficulties for conditional reasoning (beyond comprehension of the framing situations, which was controlled by the comprehension questions). It is important to note, however, that the conditionals with mathematical content were not symbolic mathematical statements, but statements about situations, which included a mathematical structure (e.g., the equivalent of different kinds of collections of red and blue gems in gold coins, or the number of windows in a “dwarf house”). We had assumed that mathematical knowledge was not primarily necessary to represent the conditionals, but to construct alternative models (e.g., different collections of red and blue gems with the same overall value). In this sense, the non-significant main effect of the conditionals’ content is in line with the rationale of the conditional reasoning tasks applied in this study.

Similar conclusions can be drawn for the interaction of conditionals’ content and grade level. Indeed, we did not find evidence of different grade-related differences of overall conditional reasoning between the two contents. This replicates the first results from our own pilot study (Datsogianni et al., 2018). Again, given our assumptions about the necessity of mathematical knowledge in our tasks, this is in line with our expectations. The missing general content difference (everyday vs. mathematical) seems to contradict results from previous studies that the conditionals’ content does play a role in conditional reasoning (Markovits and Lortie-Forgues, 2011). However, previous studies (e.g., Markovits and Vachon, 1990; Markovits and Lortie-Forgues, 2011; Markovits, 2014) compared different kinds of relations between antecedent and consequent in everyday contexts (e.g., categorical, causal, or counterfactual conditionals) that are assumed to require different levels of abstraction. Contrary to this, we used causal conditionals with EDs and corresponding conditionals with a mathematical structural mechanism mediating between antecedent and consequent. We assumed that the availability of mathematical knowledge would influence the retrieval of alternative mental models in the mathematical content condition, specifically. That we did not find general content effects seems to indicate, on first sight, that results on early conditional reasoning skills (Markovits et al., 1996) can be transferred from everyday reasoning with familiar causal conditionals to reasoning with mathematical concepts. A detailed analysis of logical forms provided a more differentiated picture.

### Definite Versus Indefinite Conclusions

Along our line of reasoning, we had expected a pronounced interaction of conditionals’ content with the type of conclusion necessary for a given inference: If mathematical knowledge was primarily necessary to construct alternative models for conditionals with mathematical content, a disadvantage should occur for those inferences that require an indefinite conclusion. Thus, we expected that the difference between performance on tasks that require a definite versus an indefinite conclusion would be more pronounced for mathematical contents. However, what we found was the opposite pattern: reasoning on indefinite forms actually turned out easier for mathematical than for everyday contents, and this effect was particularly pronounced for negatively phrased minor premises (MT vs. DA reasoning). This finding is not in line with our *a priori* predictions based on MMT, and we had no *a priori* explanation for such a result in the context of MMT. It must, however, be seen in the context of descriptively lower MT performance with mathematical, as compared to everyday contents. One reason for this pattern of results might be a stronger tendency for indefinite conclusions (“you cannot say for sure whether… or not…”) when reasoning about mathematical content on some logical forms: Negatively worded premises do not provide a specific situation (e.g., worth 15 gold coins), but only give an indication about what is *not* the case (e.g., not worth 15 gold coins). In contrast to mathematical content, it seems plausible that most students can construct specific models for everyday negation statements such as “not wet” (“dry”). Thus, for mathematical content, students might have problems to retrieve alternative models. The resulting failure to apply reasoning schemata, which they would have applied with everyday content, for conditionals with mathematical content might have weakened students’ beliefs about their answers or even lead to an increase in guessing. Indeed, the distribution of responses did not deviate from a uniform (guessing) distribution significantly for mathematical DA and MT items in grade 2. Insecurity or guessing, in turn, could be an explanation for an increased number of indefinite responses when reasoning with mathematical and negatively worded conditionals. This mechanism cannot be investigated with the data at hand, but it could be tested in future research.

Similarly, reasoning on logical forms with indefinite correct conclusions did not turn out to be significantly harder with mathematical than with everyday content for grade 2 reasoners. Again, this has to be seen in the light of a stronger tendency of second graders to give indefinite responses when reasoning about mathematical than for everyday contents. Thus, a similar mechanism as described before might explain this specific effect for grade 2, if we assume that second graders had most problems constructing alternative models to implement relevant reasoning schemata.

We had assumed that problems in alternative generation would lead to more false definite responses on the indefinite forms (AC, DA) in the mathematical context. Our findings do not support this idea. Based on the observed pattern of results, we propose that problems with generating alternatives in the mathematical context may not lead to more incorrect definite answers, but rather to more indefinite answers. Consistent with the idea that mathematical knowledge is important for conditional reasoning, when reasoning with indefinite logical forms about this kind of content, the knowledge about the conditionals’ content seemed to modulate the overall positive development of AC and DA reasoning across age found in prior studies (Janveau-Brennan and Markovits, 1999; Markovits and Barrouillet, 2002). This effect could delay the performance increase over primary school for mathematical content, but also lead to higher solution rates than with everyday contents (for which sound knowledge can be assumed) compared to MAs. Consistent with existing studies (Gauffroy and Barrouillet, 2009; Ricco, 2010; Moshman, 2011; Markovits, 2014), logical form turned out as a key factor in describing conditional reasoning performance, in this case, regarding the contrast between conditionals with mathematical and everyday contents.

The existence of (form-specific) content effects, in any case, supports the assumption that conditional reasoning is sensitive to domain differences at least in early stages of its development, as it was hypothesized in prior work (Chao and Cheng, 2000). The mechanism described above to explain a stronger tendency for indefinite conclusions for mathematical content would indicate that this domain specificity might originate from the fact that acquired reasoning schemata are generally applicable, but still dependent on domain-specific knowledge, unless they develop into more abstract reasoning schemata that work without recourse to domain-specific knowledge. However, given the unexpected pattern of results, this proposed mechanism will have to be investigated in further research. In particular, this result does not contradict the basic assumptions of MMT that the conditional inferences are derived not only from a syntactic analysis of the conditionals (based on knowledge stored in long-term memory) but also from a semantic analysis of the conditionals’ contents (Markovits et al., 1998). However, also other accounts of conditional reasoning are discussed in the literature, which could provide alternative explanations for the observed result pattern [e.g., the dual-source model of probabilistic conditional reasoning proposed^1^ by Klauer et al. (2010)].

### Positive Versus Negatively Worded Minor Premises

Regarding positive and negative wording of the minor premise, we found lower performance and a slower increase of performance on negatively phrased minor premises. Even though this wording–grade interaction was not further qualified by an interaction with the conditionals’ content in our analyses, we cannot exclude the existence of such a moderation because of the restricted power of our study for this interaction (see Supplementary Materials A.3). If these findings can be sustained with larger samples, this could indicate that the difficulties of negatively phrased minor premises, which have been mentioned in the literature (Schroyens et al., 2001) before, would not differ very strongly between everyday reasoning and the kind of reasoning with mathematical concepts we studied. Moreover, given that negatively phrased forms are investigated less frequently, the mechanisms leading to such a difference can only be hypothesized, at this point. One reason, for example, could be a difficulty representing negations in terms of mental models, which are usually assumed to represent what is *possible* under certain assumptions, not what is *impossible* (Johnson-Laird, 2001). A first indication in this direction is that our findings do not allow us to rule out the application of guessing strategies on negatively worded items in the mathematical context in grade 2 students.

### Limitations

Our study has to be considered in light of a set of limitations caused by its specific design. First, we used specific tasks to study conditional reasoning with mathematical concepts, which do not reflect deductive reasoning within a mathematical theory. We considered reasoning about mathematical structures embedded in meaningful situations to be more appropriate to study the role of mathematical concepts in conditional reasoning. Extending the results to deductive reasoning in mathematical theories, as it occurs in later years of education, however, is not straightforward. Second, our study has of course limited statistical power to identify small contrasts. A *post hoc* power analysis indicated that only contrast coefficients of up to *b* = 1.50 (*b* = 1.00 for wording of the minor premise) can be identified reliably for main effects in our setting (see Supplementary Materials A.3), which is similar to some of the observed contrast values. Insignificant findings cannot be taken as evidence for parallel developments or null effects. On the other hand, the identified differences do offer support for accounts that argue for a role of knowledge about the conditionals’ contents in conditional reasoning. Third, given the cross-sectional design, we cannot draw inferences on the individual development of primary school students’ conditional reasoning. In particular, the large number of excluded answers due to task comprehension for mathematical content items in grade 2 is an issue here. Beyond the grade-level contrasts investigated in our study, future research should also focus on individual developmental trajectories for both reasoning contents, possibly in interaction with the development of mathematical knowledge and skills. Relatedly, our study focused on the primary school age from grades 2 to 6, which is a key phase for the development of everyday conditional reasoning with causal premises. However, content effects might arise at earlier (e.g., for MP) or at later ages, when AC and DA reasoning become more secure. The latter would also correspond to complaints about problems in conditional reasoning in secondary school students (Küchemann and Hoyles, 2002). Future research could extend the current findings beyond primary school age. As for earlier ages, the availability of the required knowledge about mathematical concepts would have to be taken into account carefully, because these concepts are usually not introduced before grades 1 and 2 of primary school.

### Summary

Our results go beyond previous reports on conditional reasoning with everyday concepts and show that even elementary students are able to make valid deductions for some logical forms when reasoning about mathematical concepts. We acknowledge that there are considerable discussions among researchers about students’ ability to make conditional deductions, as well as its central importance for scientific reasoning, hypotheses generation and evaluation (Kuhn et al., 1988) and for mathematical thinking (Moshman, 1990; Markovits and Lortie-Forgues, 2011). Our results are parallel to previous results on other scientific styles (e.g., experimentation: Osterhaus et al., 2015; or probabilistic reasoning: Saffran et al., 2016) that elementary school students are able to engage in correct scientific (deductive, in this case) reasoning in specific conditions. However, our results also underpin findings that these skills may be limited to certain conditions, as MP and MT reasoning in our case, or, for example, certain kinds of covariation data in Saffran et al. (2016). In particular, understanding which factors influence students’ scientific reasoning offers starting points for evidence-based support of students’ development.

For deductive reasoning, our results provide new perspectives on the role of some knowledge about the concepts involved in the statements used for conditional reasoning. This is in line with findings that students do not use general, abstract reasoning rules at this age (Chao and Cheng, 2000). The proposed mechanism describes how knowledge about the conditional contents and more general conditional reasoning skills could interact and develop over elementary school age. In this account, weak mathematical knowledge might inhibit reasoning in forms (e.g., MT) that not only are at least partially mastered in more familiar contexts according to the literature (e.g., Markovits, 2000; Markovits and Thompson, 2008), but also lead to more correct answers on other logical forms (e.g., DA). Our study provides indications that with increasing familiarity with mathematical concepts in higher grades, performance at least in reasoning with definite forms (MP and MT) on mathematical concepts approaches performance in everyday contexts. All in all, our results are still in line with a model that puts both mathematical knowledge and conditional reasoning strategies as necessary and mutually non-compensating prerequisites of conditional reasoning with mathematical concepts.

If mathematical knowledge is necessary for conditional reasoning with these concepts, it is an open question if this connection can be used in the other direction: Experiencing conditional inferences with mathematical concepts and discussing alternatives as well as other models for the involved conditionals might not only help to increase conditional reasoning skills, but also add to students’ knowledge about these concepts. This is in line with current standards (National Governors Association Center for Best Practices, and Council of Chief State School Officers, 2010): argumentation, proof, and reasoning should be incorporated regularly into the mathematics classroom from prekindergarten through grade 12.

## Data Availability Statement

The datasets generated for this study are available on request to the corresponding author.

## Ethics Statement

The studies involving human participants were reviewed and approved by Centre of Educational Research and Evaluation of Cyprus Pedagogical Institute and Cyprus Ministry of Education and Culture. Written informed consent to participate in this study was provided by the participants’ legal guardian/next of kin.

## Author Contributions

AD, SU, and BS contributed to the design of the study. AD collected all data. AD, BS, HM, and SU discussed the statistical analysis. AD and SU performed the statistical analysis. AD wrote the first draft of the manuscript. SU revised parts of the manuscript. BS and HM provided feedback to the original manuscript and its revisions. All authors contributed to manuscript revision, read and approved the submitted version.

## Conflict of Interest

The authors declare that the research was conducted in the absence of any commercial or financial relationships that could be construed as a potential conflict of interest.

## Footnotes

1 According to this model, inferences are based on two sources of evidence: logical form (decontextualized source of evidence) and prior knowledge about the reasoning context.
