# Supplementary material for: Reasoning With Conditionals About Everyday and Mathematical Concepts in Primary School
Source: Front Psychol. 2020 Oct 29;11:531640. doi: 10.3389/fpsyg.2020.531640 (PMC7658316; doi:10.3389/fpsyg.2020.531640)
Supplement: Supplementary file 1 [file Data_Sheet_1.docx]

# Supplementary Materials

## Examples of conditionals for both content types

| **Everyday content**  *Major premise:* If someone’s finger is cut deeply while cooking, then it bleeds. | | |
| --- | --- | --- |
| *Logical form* | *Minor premise* | *Correct conclusion* |
| MP | Peter’s finger is cut deeply while cooking. | Peter’s finger is bleeding. |
| MT | Peter’s finger is not bleeding. | Peter’s finger was not cut deeply while cooking. |
| AC | Peter’s finger is bleeding. | One cannot say whether Peter’s finger was cut deeply while cooking, or not. |
| DA | Peter did not cut his finger deeply while cooking. | One cannot say whether Peter’s finger is bleeding, or not. |

## Examples of conditional inferences with both content types

Table S1: Examples of conditional inferences with everyday content.

| **Mathematical content**  *Major premise:* If the box contains exactly 2 blue diamonds and 3 red diamonds, then the diamonds in the box are worth 12 gold coins. | | |
| --- | --- | --- |
| *Logical form* | *Minor premise* | *Correct conclusion* |
| MP | Peter’s box contains exactly 2 blue diamonds and 3 red diamonds. | The diamonds in Peter’s box are worth 12 gold coins. |
| MT | The diamonds in Peter’s box are not worth 12 gold coins. | Peter’s box does not contain exactly 2 blue diamonds and 3 red diamonds. |
| AC | The diamonds in Peter’s box are worth 12 gold coins. | One cannot say whether Peter’s box contains exactly 2 blue diamonds and 3 red diamonds. |
| DA | Peter’s box does not contain exactly 2 blue diamonds and 3 red diamonds. | One cannot say whether the diamonds in Peter’s box are worth exactly 12 gold coins, or not. |

Table S2: Examples of conditional inferences with everyday content.

## Model selection

| *nr* | *formula*  *decision made on the model* | *chi-square difference test* |
| --- | --- | --- |
| 1 | 1 + working-memory +  grade*content*wording*conclusion +  (1 + grade + wording + conclusion \| conditional) +  (1 + content*wording*conclusion \| person) | Singular model fit. |
|  | Removed random slopes for interaction effects over person. |  |
| 2 | 1 + working-memory +  grade*content* wording*conclusion +  (1 + grade + wording + conclusion \| conditional) +  (1 + content + wording + conclusion \| person) |  |
|  | Removed random slope grade \| conditional. | *χ*^2^(9) = 9.24, *p* = .42 |
| 3 | 1 + working.memory +  grade*content*wording*conclusion +  (1 + wording + conclusion \| conditional) +  (1 + content + wording + conclusion \| person) |  |
|  | Removed non-significant interactions. | *χ*^2^(6) = 6.44, *p* = .38 |
| 4 | 1 + working-memory +  grade*wording +  grade*content*conclusion +  content*wording*conclusion +  (1 + wording + conclusion \| conditional) +  (1 + content + wording + conclusion \| person) |  |

Table S3: Models considered during model selection, including stepwise chi-square difference tests and R formulas (lme4). Interaction terms include all corresponding main effects and lower-order interactions.

## Power analysis

A post-hoc (sensitivity) power analysis for the contrast coefficients corresponding to the main effects of grade level (contrasts between grade 2 and grade 4 resp. between grade 2 and grade 6), content (everyday vs. mathematical), wording of minor premise (positive vs. negative), and type of conclusion (definite vs. indefinite) was performed based on the final estimated model that is presented in the manuscript. Moreover, the power of the grade-level x wording and the grade level x content x conclusion type interactions are relevant for the discussion section. Thus, similar power analyses were calculated for the corresponding interaction effect coefficients.

The package simr was used to perform a simulation-based power analysis with four different contrast strengths (*|B|* ∈ {*0.5, 1.0, 1.5, 2.0*}) and N = 50 simulations for each contrast strength.

|  | *\|B\|* | | | |
| --- | --- | --- | --- | --- |
| *contrast* | *0.50* | *1.00* | *1.50* | *2.00* |
| wording of minor premise (positive vs. negative) | .36 | **.80** | **.98** | **1.00** |
| type of conclusion (definite vs. indefinite) | .22 | .62 | **.94** | **1.00** |
| content (everyday vs. mathematical) | .20 | .64 | **.86** | **1.00** |
| grade level (grade 2 vs. grade 4) | .38 | **.86** | **1.00** | **1.00** |
| grade level (grade 2 vs. grade 6) | .42 | **.82** | **.94** | **1.00** |
| grade level (grade 2 vs. grade 4) x  content x  type of conclusion | .14 | .40 | .62 | **.96** |
| grade level (grade 2 vs. grade 6) x  content x  type of conclusion | .32 | .44 | .72 | **.92** |
| grade level (grade 2 vs. grade 4) x  wording of minor premise | .48 | **.92** | **1.00** | **1.00** |
| grade level (grade 2 vs. grade 6) x  wording of minor premise | .42 | **.96** | **1.00** | **1.00** |

Table S4: Estimated power for contrasts of main effects and different contrast strengths. Power estimates above .80 are set in bold.

## Interview protocol

### Training of the alternative answers

One red and one green marble are in a small open box from the start, both visible for the child. The researcher points to the red marble when explaining the game stating that all questions will be about the red marble, because she/he likes it most.

I will show you something with these marbles and ask some questions. This card can help you regarding the possible answers (the researcher shows the relevant visualization) The researcher holds up a red marble that the child can see and then hides it in her/his right hand.

What can you say for sure about the red marble?

Possible answers (the researcher points the three alternative answers while reading them)

It is certain that the red marble is in this hand.
It is certain that the red marble is not in this hand.
It is uncertain. One cannot say for sure whether the red marble is in this hand or not.

If the child answers “is in this hand”, the researcher points the “check” of the visualization (in case that the child did not already do that).
Otherwise: You saw that I took the red marble with my hand, so you can say for sure that the red marble is in this hand. Researcher points to the “yes, certain” option (check).

Then the researcher holds up only the green marble that the child can see and then hides it in her/his right hand. Then the unused red marble remains in the container.
What can you say for sure about the red marble?

Possible answers (the researcher points the three alternative answers while reading them)

It is certain that the red marble is in this hand.
It is certain that the red marble is not in this hand.
It is uncertain. One cannot say for sure whether the red marble is in this hand or not.

If the child answers “is not in this hand”, the researcher points the “cross” of the visualization (in case that the child did not already do that).
Otherwise: You saw that I took the green marble with my hand, so you can say for sure that the red marble is not in this hand. Researcher points to the “no, certain” option (cross).

Then the researcher puts both marbles into her right hand, and then shuffles them between both hands, so that one is in the right and one is in the left hand. The researcher holds closed the right hand and asks the following question:
What can you say for sure about the red marble?

Possible answers (the researcher points the three alternative answers while reading them)

It is certain that the red marble is on this hand.
It is certain that the red marble is not this hand.
It is uncertain. One cannot say for sure whether the red marble is in this hand or not.

If the answer is right, the researcher points the “question mark” of the visualization (in case that the child did not already do that).
If the child tries to guess which marble is hidden and gives a random wrong reply, the researcher asks whether the child can be really sure about its answer or not and gives the explicit answer. Can you be really sure about that? In some cases you cannot be really sure about the answer and this is true. The researcher points the “question mark” of the visualization.

### Conditional reasoning tasks with mathematical content

#### Introducing Peter

First of all, we should get to know a person who reveals some truths in our stories. That person is Peter, a Little Scientist! In our stories, we assume that Peter always tells the truth. He is always curious! Let’s try to help him to answer some more questions!

#### Mathematical tasks-Introduction

Peter is on a discovery journey with some little explorers to a small and hidden Island now which is inhabited by dwarfs

#### Exploring the pirate boat

Peter is walking with the little explorers and they just found some treasure boxes. We know that the boxes contain some blue and red diamonds.
Peter has found a message in which the value of each diamond in gold coins is indicated
(picture of the message which is shown during the task below):
- the blue diamond is worth 3 gold coins
- the red diamond is worth 2 gold coins

#### Control question

For instance, in a treasure box there is 1 blue diamond and 2 red diamonds. How many gold coins is this worth?

#### Task M1 (treasure box 1)

So, Peter afterwards found out something (additional picture which is shown during the task):
If the box contains exactly 2 blue diamonds and 3 red diamonds, then the diamonds in the box are worth 12 gold coins.
It is certain that this is really true.
I will ask you some questions about this; please think very well before answering them.

#### M1.1 (MP)

This is Maria. Her box contains exactly 2 blue diamonds and 3 red diamonds. Based on what he knows, what can Peter say for sure?
The researcher points the three alternative answers while reading them:

a)  The diamonds in the box are worth 12 gold coins.
b)  The diamonds in the box are not worth 12 gold coins
c)  He cannot be sure whether the diamonds in the box are worth 12 gold coins or not.

#### M1.2 (MT)

This is Stelios. The diamonds in his box are not worth 12 gold coins. Based on what he knows, what can Peter say for sure?
The researcher points the three alternative answers while reading them:

a)  The box contains exactly 2 blue diamonds and 3 red diamonds.
b)  The box does not contain exactly 2 blue diamonds and 3 red diamonds.
c)  He cannot be sure whether the box contains exactly 2 blue diamonds and 3 red diamonds or not.

#### M1.3 (DA)

The little explorer Helen has found a box that is not one that contains exactly 2 blue diamonds and 3 red diamonds.
Based on what he knows, what can Peter say for sure?
The researcher points the three alternative answers while reading them:

a)  The diamonds in the box are worth 12 gold coins.
b)  The diamonds in the box are not worth 12 gold coins
c)  He cannot be sure whether the diamonds in the box are worth 12 gold coins or not.

#### M1.4 (AC)

This is Charis. The diamonds in his box are worth 12 gold coins. Based on what he knows, what can Peter say for sure?
The researcher points the three alternative answers while reading them:

a)  The box contains exactly 2 blue diamonds and 3 red diamonds.
b)  The box does not contain exactly 2 blue diamonds and 3 red diamonds.
c)  He cannot be sure whether the box contains exactly 2 blue diamonds and 3 red diamonds or not.

#### Task M2 (treasure box 3)

Peter has just found out something about the treasure boxes. Let’s see what he found out:
If the box contains exactly 4 blue diamonds and 1 red diamond, then the diamonds in the box are worth 14 gold coins.
It is certain that this is really true.
It is certain that this is really true.

#### M2.1 (MT)

This is Charis. The diamonds in his second box are not worth 14 gold coins. Based on what he knows, what can Peter say for sure?
The researcher points the three alternative answers while reading them:

a)  The box contains exactly 4 blue diamonds and 1 red diamond.
b)  The box does not contain exactly 4 blue diamonds and 1 red diamond.
c)  He cannot be sure whether the box contains exactly 4 blue diamonds and 1 red diamond or not.

#### M2.2 (AC)

This is Stelios. The diamonds in his second box are worth 14 gold coins. Based on what he knows, what can Peter say for sure?
The researcher points the three alternative answers while reading them:

a)  The box contains exactly 4 blue diamonds and 1 red diamond.
b)  The box does not contain exactly 4 blue diamonds and 1 red diamond.
c)  He cannot be sure whether the box contains exactly 4 blue diamonds and 1 red diamond or not.

#### M2.3 (MP)

This is Helen. Her second box contains exactly 4 blue diamonds and 1 red diamond. Based on what he knows, what can Peter say for sure?
The researcher points the three alternative answers while reading them:

a)The diamonds in the box are worth 14 gold coins.
b)  The diamonds in the box are not worth 14 gold coins
c)  He cannot be sure whether the diamonds in the box are worth 14 gold coins or not.

#### M2.4 (DA)

This is Maria. Her second box does not contain exactly 4 blue diamonds and 1 red diamond. Based on what he knows, what can Peter say for sure?
The researcher points the three alternative answers while reading them:

a)The diamonds in the box are worth 14 gold coins.
b)  The diamonds in the box are not worth 14 gold coins
c)  He cannot be sure whether the diamonds in the box are worth 14 gold coins or not.

#### Task M3 (Dwarf house windows)

Peter is at this island and observes dwarfs’ houses.
Dwarfs build their houses so that there are rooms which all have this form:

The houses always have one or more rows of rooms which are all equally long. For example, dwarfs may have a house like this:

They also make one window on each exterior wall of each room.

6 windows example:

#### Control question

A dwarf house has 2 rows with 2 rooms each. How many windows does it have?

#### Instruction

Peter tries to find out as much as possible about houses of Dwarfs! So Peter has found out something (additional picture which is shown during the task below):
If a dwarf house has exactly 2 rows of 5 rooms each, then it has 14 windows.
It is certain that this is really true.
I will ask you some questions about this; please think very well before answering them.

#### M3.2 (DA)

This is one of the “bashful dwarfs”. Their house is not one that has exactly 2 rows with 5 rooms in each row. Based on what he knows, what can Peter say for sure?
The researcher points the three alternative answers while reading them:

a) The house has 14 windows.
b)  The house does not have 14 windows.
c)  He cannot be sure whether the house has 14 windows or not.

#### M3.2 (AC)

This is one of the “happy dwarfs”. Their house is one that has exactly 14 windows. Based on what he knows, what can Peter say for sure?
The researcher points the three alternative answers while reading them:

a)  The house has exactly 2 rows with 5 rooms in each row.
b)  The house does not have exactly 2 rows with 5 rooms in each row.
c)  He cannot be sure whether the house 2 rows with 5 rooms in each row or not.

#### M3.3 (MP)

This is one of the “sneezy dwarfs”. Their house has exactly 2 rows of 5 rooms each.
Based on what he knows, what can Peter say for sure? The researcher points the three alternative answers while reading them:

a) The house has 14 windows.
b)  The house does not have 14 windows.
c)  He cannot be sure whether the house has 14 windows or not.

#### M3.4 (MT)

This is one of the “grumpy dwarfs”. Their house is not one that has exactly 14 windows. Based on what he knows, what can Peter say for sure?
The researcher points the three alternative answers while reading them:

a)  The house has exactly 2 rows with 5 rooms in each row.
b)  The house does not have exactly 2 rows with 5 rooms in each row.
c)  He cannot be sure whether the house 2 rows with 5 rooms in each row or not.

#### Task M4 (dwarf house rooms)

Peter is at this island and observes dwarfs’ houses.

#### Comprehension question

There is a dwarfs’ house which has 2 rows of 3 rooms each, how many rooms does it have?

#### Instruction

Peter has found out a second rule about the dwarfs’ houses in that village:
If a dwarf house has exactly 2 rows of 6 rooms each, then it has 12 rooms.
It is certain that this is really true.
I will ask you some questions about this; please think very well before answering them.

#### M4.1 (MP)

This is one of the “sleepy dwarfs”. Their house has exactly 2 rows of 6 rooms each. Based on what he knows, what can Peter say for sure?
The researcher points the three alternative answers while reading them:

a)  The house has exactly 12 rooms.
b)  The house does not have exactly 12 rooms.
c)  He cannot be sure whether the house has exactly 12 rooms or not.

#### M4.2 (DA)

This is one of the “wise dwarfs”. Their house is not one that has exactly 2 rows with 6 rooms in each row. Based on what he knows, what can Peter say for sure?
The researcher points the three alternative answers while reading them:

a)  The house has exactly 12 rooms.
b)  The house does not have exactly 12 rooms.
c)  He cannot be sure whether the house has exactly 12 rooms or not.

#### M4.3 (AC)

This is one of the “dopey dwarfs”. Their house is not one that has exactly 12 rooms. Based on what he knows, what can Peter say for sure?
The researcher points the three alternative answers while reading them:

a)  The house has exactly 2 rows with 6 rooms in each row.
b)  The house does not have exactly 2 rows with 6 rooms in each row.
c)  He cannot be sure whether the house has exactly 2 rows with 6 rooms in each row or not.

#### M4.4 (MT)

This is one of the “kind dwarfs”. Their house has exactly 12 rooms. Based on what he knows, what can Peter say for sure?
The researcher points the three alternative answers while reading them:

a)  The house has exactly 2 rows with 6 rooms in each row.
b)  The house does not have exactly 2 rows with 6 rooms in each row.
c)  He cannot be sure whether the house has exactly 2 rows with 6 rooms in each row or not.

### Conditional reasoning tasks with everyday content

### Task E1 (dropping the glass)

Peter is in his house now. At his home, there are some glasses in the kitchen. Sometimes it happens that a glass is dropped to the ground in the kitchen. Peter has found out that (additional picture which is shown during the task below):
If a glass is dropped on the ground in the kitchen, then there is a sound.
It is certain that this is really true.
I will ask you some questions about this; please think very well before answering them.

#### E1.1 (AC)

Peter tries to get some rest in his bedroom. There is a sound now. Based on what he knows, what can Peter say for sure?
The researcher points the three alternative answers while reading them:

a)  A glass is dropped on the ground in the kitchen.
b)  No glass is dropped on the ground in the kitchen
c)  He cannot be sure whether a glass is dropped on the ground in the kitchen or not.

#### E1.2 (DA)

Peter is in the kitchen, observing. No glass is dropped on the ground in the kitchen now. Based on what he knows, what can Peter say for sure?
The researcher points the three alternative answers while reading them:

a)  There is a sound now.
b)  There is no sound now.
c)  He cannot be sure whether there is a sound or not.

#### E1.3 (MT)

Peter tries to get some rest in his bedroom. There is no sound now. Based on what he knows, what can Peter say for sure?
The researcher points the three alternative answers while reading them:

a)  A glass is dropped on the ground in the kitchen.
b)  No glass is dropped on the ground in the kitchen
c)  He cannot be sure whether a glass is dropped on the ground in the kitchen or not.

#### E1.4 (MP)

Peter is in the kitchen, observing. A glass is dropped on the ground in the kitchen now.
Based on what he knows, what can Peter say for sure?
The researcher points the three alternative answers while reading them:

a)  There is a sound now.
b)  There is no sound now.
c)  He cannot be sure whether there is a sound or not.

#### Task E2 (cooking)

Peter sometimes helps his parents during the cooking. For example, he cuts fruits with a knife.
Peter has found out something (additional picture which is shown during the task below):
If someone’s finger is cut deeply while cooking, then it bleeds.
It is certain that this is really true.
I will make some questions; please think very well before answering them.

#### E2.1 (MT)

Peter visited his friend, George. George’s finger is not bleeding. Based on what he knows, what can Peter say for sure?
The researcher points the three alternative answers while reading them:

a) George’s finger has just been cut deeply while cooking.
b) George’s finger has not just been cut deeply while cooking.
c) He cannot be sure whether George’s finger has just been cut deeply while cooking or not.

#### E2.2 (MP)

Peter has been told that Cathrin’s finger has just been cut deeply while cooking. Based on what he knows, what can Peter say for sure?
The researcher points the three alternative answers while reading them:

a) Cathrin’s finger is bleeding.
b) Cathrin’s finger is not bleeding.
c) He cannot be sure whether Cathrin’s finger is bleeding or not.

#### E2.3 (AC)

Peter visited his friend, Despina. Despina’s finger is bleeding. Based on what he knows, what can Peter say for sure?
The researcher points the three alternative answers while reading them:

a) Despina’s finger has just been cut deeply while cooking.
b) Despina’s finger has not just been cut deeply while cooking.
c) He cannot be sure whether Despina’s finger has just been cut deeply while cooking or not.

#### E2.3 (DA)

Peter has been told that Kosta’s finger has not just been cut deeply while cooking. Based on what he knows, what can Peter say for sure?
The researcher points the three alternative answers while reading them:

a) Kosta’s finger is bleeding.
b) Kosta’s finger is not bleeding.
c) He cannot be sure whether Kosta’s finger is bleeding or not.

*Task E3 (swimming pool)*

Peter really likes swimming pools as he loves swimming. So, Peter has found out something (additional picture which is shown during the task below):
If someone jumps into a swimming pool, then she or he gets wet.
It is certain that this is really true.
I will ask you some questions about this; please think very well before answering them.

#### E3.1 (AC)

A few days later, Peter just arrived at the swimming pool. His friend Vivi is wet now. Based on what he knows, what can Peter say for sure?
The researcher points the three alternative answers while reading them:

a)  Vivi has jumped into the swimming pool.
b)  Vivi has not jumped into the swimming pool.
c)  He cannot be sure whether Vivi has jumped into the swimming pool or not.

#### E3.2 (MT)

Peter just arrived at the swimming pool. His friend Sakis is not wet now. Based on what he knows, what can Peter say for sure?
The researcher points the three alternative answers while reading them:

a)  Sakis has jumped into the swimming pool.
b)  Sakis has not jumped into the swimming pool.
c)  He cannot be sure whether Sakis has jumped into the swimming pool or not.

#### E3.3 (MP)

Peter has been told that his friend, Mary, has just jumped into the swimming pool. Based on what he knows, what can Peter say for sure?
The researcher points the three alternative answers while reading them:

a) Mary is wet now.
b) Mary is not wet now.
c) He cannot be sure whether Mary is wet or not.

#### E3.4 (DA)

Peter has been told that his friend, Dimitris, has not just jumped into the swimming pool. Based on what he knows, what can Peter say for sure?
The researcher points the three alternative answers while reading them:

a) Dimitris is wet now.
b) Dimitris is not wet now.
c) He cannot be sure whether Dimitris is wet or not.

#### Task E4 (broken arm)

Peter has found out that: (additional picture which is shown during the task below):
If someone breaks his arm, then she or he hurts.
It is certain that this is really true.
I will ask you some questions about this; please think very well before answering them.

#### E4.1 (DA)

Peter has been told that Chris has not just broken his arm. Based on what he knows, what can Peter say for sure?
The researcher points the three alternative answers while reading them:

a) Chris’ arm hurts now.
b)  Chris’ arm does not hurt now.
c)  He cannot be sure whether Chris’ arm hurts now or not.

#### E4.2 (AC)

Peter visited his friend, Ntina in order to see her. Ntinas arm hurts now. Based on what he knows, what can Peter say for sure?
The researcher points the three alternative answers while reading them:

a)  Ntina has just broken her arm.
b)  Ntina has not just broken her arm.
c)  He cannot be sure whether Ntina has just broken her arm or not.

#### E4.3 (MT)

Peter visited his friend, Stella. Stellas arm does not hurt now.
Based on what he knows, what can Peter say for sure?
The researcher points the three alternative answers while reading them:

a)  Stellas has just broken her arm.
b)  Stellas has not just broken her arm.
c)  He cannot be sure whether Stellas has just broken her arm or not.

#### E4.4 (MP)

Peter has been told that his friend, Nektaria, has just broken her arm. Based on what he knows, what can Peter say for sure?
The researcher points the three alternative answers while reading them:

a) Nektarias arm hurts now.
b)  Nektarias arm does not hurt now.
c)  He cannot be sure whether Nektarias arm hurts now or not.
